# Implementation of hydroponic teaching and research platforms at primarily undergraduate institutions

**DOI:** 10.3389/feduc.2026.1808562

**Published:** 2026-06-18

**Authors:** Annie Wang, Brooke Duncan, Emily Duncan, Eli Johnson, Kevin Wang, Byron Meade

**Affiliations:** Division of Math and Natural Sciences, University of Pikeville, Pikeville, KY, United States

**Keywords:** controlled environment agriculture, cure, experiential learning, hydroponics, plant biology education, primarily undergraduate institutions, stem education, undergraduate research

## Abstract

Many primarily undergraduate institutions (PUIs) offer coursework in plant biology, agriculture, and environmental sciences but lack dedicated plant growth facilities and controlled-environment infrastructure. Hydroponic systems provide an indoor platform for year-round plant cultivation, hypothesis-driven experimentation, and integration with course-based undergraduate research experiences (CUREs). At our institution, commercial and custom-built hydroponic systems were integrated into existing teaching laboratories and maintained by faculty, laboratory staff, and undergraduate work-study students. This structure supported consistent growth conditions, repeated student-led experimentation, and practical training in plant cultivation and biotechnology. Based primarily on this institutional experience, this mini-review synthesizes pedagogical outcomes, implementation onsiderations, and funding pathways, highlighting hydroponic platforms as effective infrastructure for experiential, research-centered STEM education at PUIs.

## 1 Introduction

Primarily undergraduate institutions (PUIs) offering plant biology, botany, agriculture, and environmental science courses often face structural constraints that limit modern experimental instruction. Limited access to greenhouses, growth chambers, and controlled-environment facilities can restrict research-oriented plant science training (Mayfield-Smith et al., 2021). As a result, plant-focused instruction often relies on outdoor observations, field trips, preserved specimens, or short-term demonstrations. These approaches provide valuable exposure to biodiversity and natural systems (Çetin, 2025), but they are less suited for controlled experimentation, reproducible data collection, and year-round student-led research, especially when seasonal constrains limit plant growth (Almerino and Peria, 2024).

To address these challenges, we implemented hydroponic systems within existing laboratory spaces as a platform for undergraduate teaching and research. Hydroponics enables indoor, soil-free cultivation under controlled nutrient, light, and environmental conditions, offering a practical alternative to greenhouse-dependent instruction (Almerino and Peria, 2024; Cummings and Cummings, 2021; Lakomy et al., 2024; Perez et al., 2024; Ranade and Chaudhari, 2026; Wang et al., 2025). In our experience, this approach supported hands-on experimentation, reproducible data collection, and sustained student engagement across academic terms.

When embedded in undergraduate curricula, hydroponic systems can serve as both instructional tools and infrastructure for CUREs and workforce preparation in plant and environmental sciences (Khastini and Maryani, 2025). This mini-review is grounded primarily in our institutional implementation and uses that experience to examine pedagogical benefits, implementation strategies, assessment considerations, transferability, and funding pathways relevant to PUIs. Its scope is educational implementation rather than a comprehensive global survey of hydroponic technologies, with emphasis on institutions limited by greenhouse access, laboratory space, and seasonal constraints.

## 2 Advantages of hydroponic implementation in PUI plant sciences

Hydroponic platforms provide a scalable way to integrate year-round plant experimentation, undergraduate research, and inquiry-based instruction into PUI curricula without greenhouse infrastructure (Corveleyn et al., 2020; Cummings and Cummings, 2021; Khastini and Maryani, 2025; Lakomy et al., 2024; Pennisi et al., 2025).

Compared with field-based instruction, hydroponic platforms allow greater control over nutrient composition, light, water availability, and growth duration. Field trips and outdoor observations remain valuable for biodiversity and ecological learning, but they are often limited by seasonality, weather, transportation, and lower experimental reproducibility. Hydroponics therefore complements field-based learning by enabling repeated, student-led experiments and quantitative data collection within a single academic term.

### 2.1 Enhancing student engagement through familiar plant Species

Student engagement is central to active and experiential STEM education (Bennie et al., 2025; Driessen et al., 2020; Reeves et al., 2024; Wang et al., 2025). In PUI plant science courses, students are often motivated by growing familiar species, such as vegetables, herbs, fruits, and ornamental flowers. These recognizable plants help connect abstract biological concepts to everyday experience and strengthen cognitive engagement (Cummings and Cummings, 2021; Krosnick and Moore, 2025; Lakomy et al., 2024) (Table 1).

**FIGURE 1:**
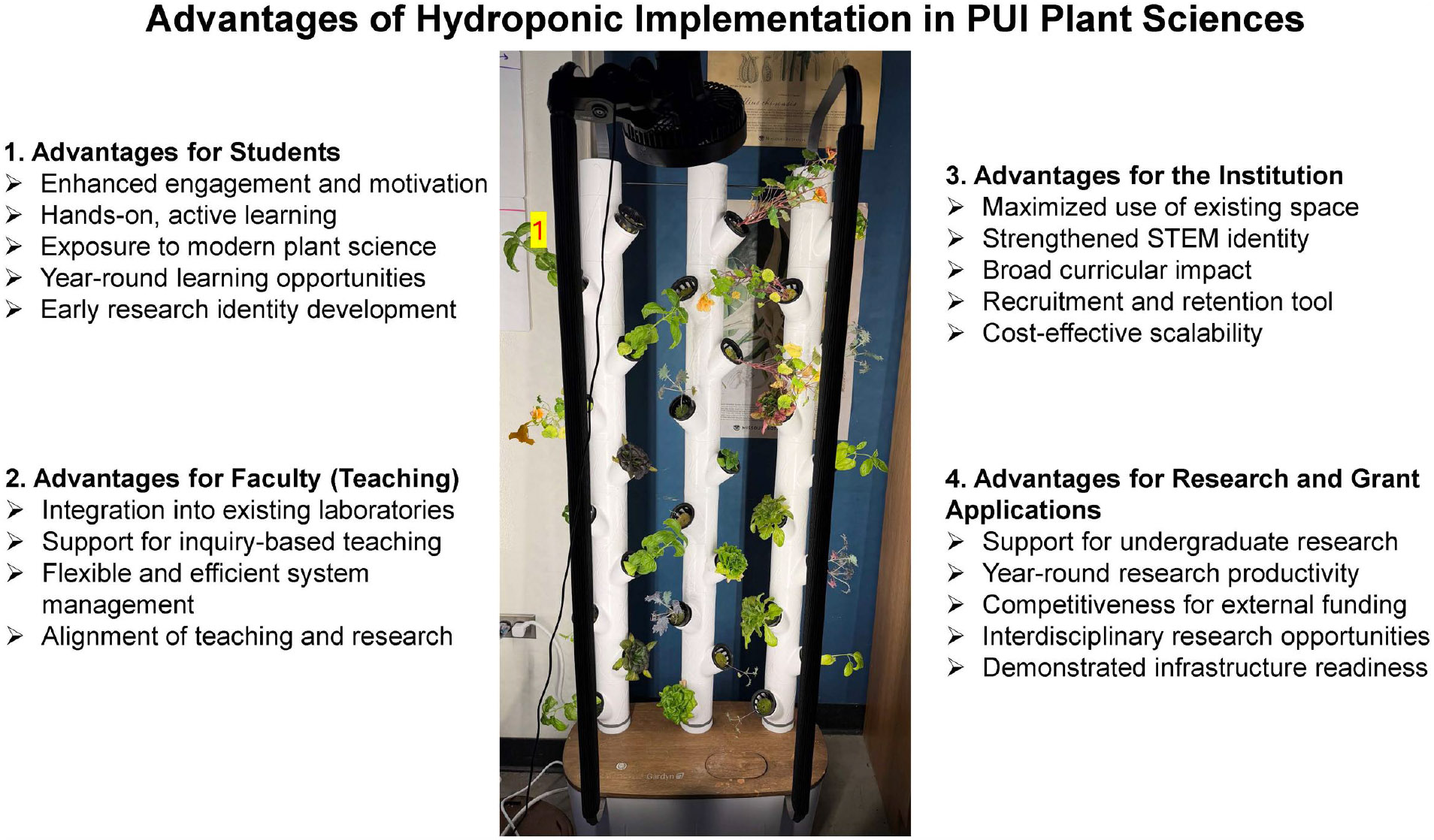
Hydroponic teaching and research platform implemented at a primarily undergraduate institution. A compact, vertically oriented hydroponic system implemented in a botany teaching laboratory at the University of Pikeville, a primarily undergraduate institution (PUI). The system is integrated into an existing biology laboratory and operates without the need for a greenhouse or dedicated plant growth facility. Undergraduate students use the platform to germinate, cultivate, and monitor familiar plant species under controlled conditions, with plants grown simultaneously at multiple developmental stages from seed germination to vegetative growth and flowering within approximately 1–3 weeks. Labeled plant species include: (1) Lettuce (*Lactuca sativa*), (2) Basil (*Ocimum basilicum*), (3) Kale (*Brassica oleracea* var. *acephala*), (4) Nasturtium (*Tropaeolum majus*; flowering), and (5) Pac Choi (*Brassica rapa* subsp. *chinensis*).

Hydroponic systems support this engagement by combining flexible plant selection with controlled and reproducible growth conditions (Reza et al., 2025). Allowing students to select and cultivate species increase student agency, autonomy, and ownership of learning (Almerino and Peria, 2024) (Figure 1). Compared with field-based instruction, hydroponics also supports rapid germination, visible phenotypic responses, and short experimental cycles, enabling formative assessment and repeated hypothesis testing within one semester (Cummings and Cummings, 2021; Lakomy et al., 2024).

### 2.2 Motivating undergraduate research interests and career pathways

Hydroponic platforms can also motivate undergraduate research participation and career exploration. By working with familiar plant species, students encounter a lower barriers to experimental design, data collection, and data interpretation. Measurable growth responses and reproducible datasets can promote ownership of projects and strengthen scientific identity (Cooper et al., 2020).

Controlled-environment cultivation further exposes students to areas beyond traditional field-based plant studies, including controlled-environment agriculture, plant biotechnology, and sustainable food systems (Wang et al., 2025). For many PUI students, these experiences provide an early introduction to modern plant science research and may encourage advanced coursework, mentored research, graduate study, or careers in agriculture, biotechnology, and environmental sciences.

### 2.3 Supporting faculty teaching and research at PUIs

**FIGURE 2:**
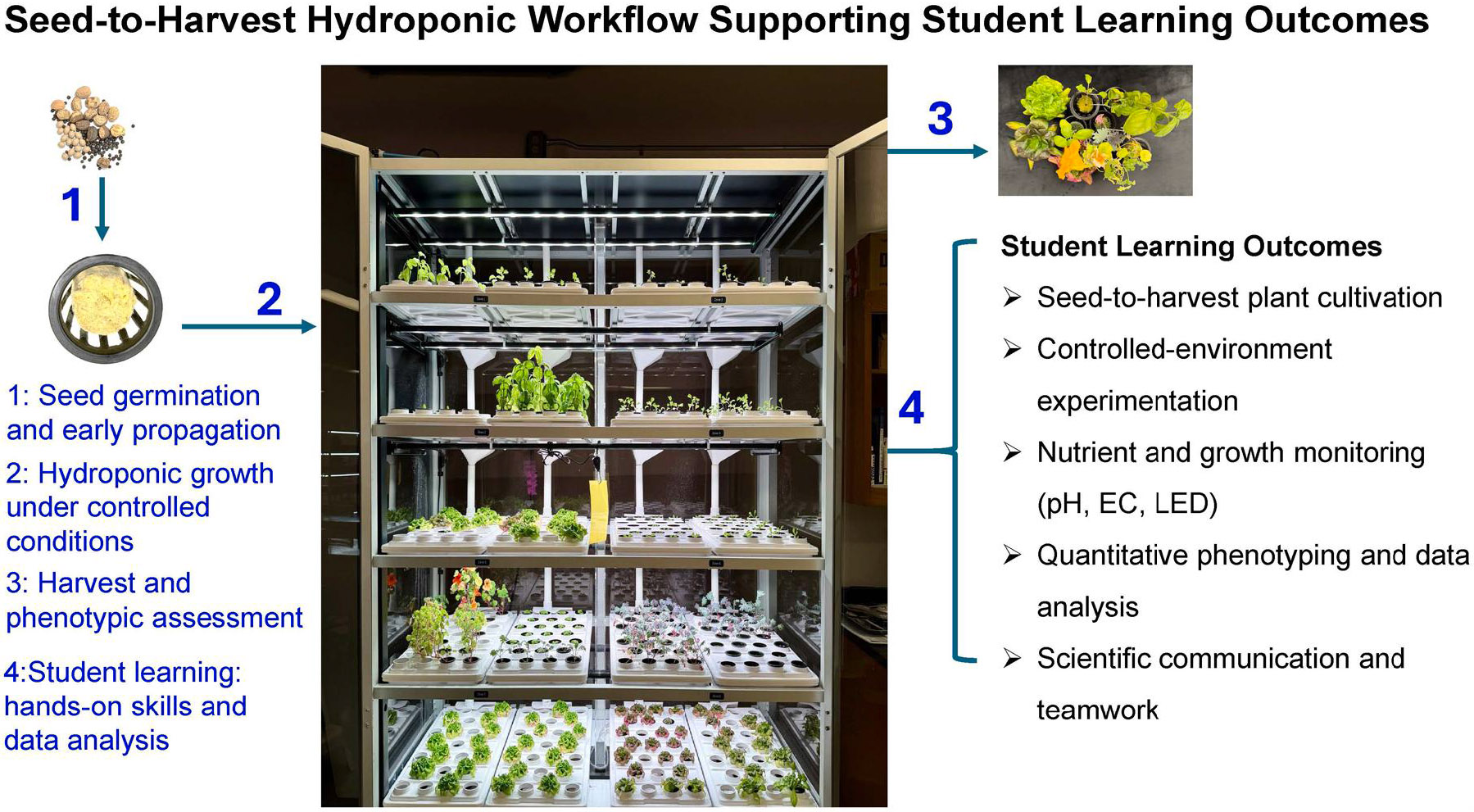
Seed-to-harvest hydroponic workflow supporting student learning outcomes. Seed-to-harvest hydroponic workflow used to support hands-on learning in undergraduate plant biology and botany courses at a primarily undergraduate institution (PUI). Students engage in seed germination and early propagation (1), hydroponic growth under controlled environmental conditions (2), and harvest with phenotypic assessment (3), leading to student learning outcomes that include experimental design, nutrient and growth monitoring (pH, EC, LED lighting), quantitative phenotyping, data analysis, scientific communication, and teamwork (4).

For faculty at PUIs, hydroponic platforms support active plant science instruction while remaining feasible within space, budget, and teaching-load constraints (Almerino and Peria, 2024; Boyd et al., 2025). Compact, modular systems can be installed in standard biology or botany laboratories without greenhouse space or renovation (Choi et al., 2025; Velazquez-Gonzalez et al., 2022) (Figures 1, 2), allowing faculty to use exiting resources for plant-based experimentation (Cummings and Cummings, 2021).

Because hydroponic systems operate year-round, they support inquiry-based laboratories, CUREs (Gibbons Johnson 2025; Wang et al., 2025), and multi-week experiments aligned with academic calendars. They also enhance faculty research capacity by enabling continuous plant growth, reproducible data collection, undergraduate mentoring, and integration with plant physiology, biotechnology, and environmental science workflows (Cummings and Cummings, 2021; Grünhofer et al., 2021; Heuvelink et al., 2025; Khastini and Maryani, 2025; Lakomy et al., 2024).

### 2.4 Institutional and research advantages

At the institutional level, hydroponic systems make efficient use of existing laboratory space and can serve programs in plant biology, botany, agriculture, environmental science, and biotechnology. Their visibility and interdisciplinary relevance strengthen institutional STEM identity, support recruitment and retention, and demonstrate commitment to experiential learning.

Hydroponic platforms can also strengthen competitiveness for external funding by aligning with priorities in undergraduate research, CURE implementation, workforce development, sustainability, and PUI capacity building. They fit education- and workforce-focused opportunities from NSF programs supporting undergraduate research and infrastructure, USDA education and, NIH mechanisms emphasizing undergraduate research integration at PUIs. An operational hydroponic system provides tangible evidence of infrastructure readiness, feasibility, and interdisciplinary potential (Wu et al., 2025).

Collectively, these benefits show how hydroponic platforms can connect student engagement, CURE-based learning, faculty research capacity, institutional infrastructure, and funding competitiveness. Rather than functioning only as classroom demonstrations, they provide an integrated model for strengthening undergraduate STEM education at PUIs. The following section illustrates this model through our institutional implementation at a rural PUI.

## 3 Implementation strategies for hydroponic platforms at primarily undergraduate institutions

### 3.1 Institutional context and rationale

The University of Pikeville (UPIKE) is a rural PUI in eastern Kentucky within Central Appalachia. Like many rural PUIs, UPIKE lacks a greenhouse, garden space, or dedicated controlled-environment plant growth facility. Plant biology and botany instruction therefore relied largely on outdoor field activities, preserved specimens, or short-term potted-plants demontration (Çetin 2025; McDonough MacKenzie et al., 2020), which provide useful exposure to plant diversity but limited opportunities for controlled, reproducible, and sustained inquiry.

To address these constraints, Dr. Byron Meade (corresponding author) led the integration of indoor hydroponic systems as a complementary platform for undergraduate teaching and research. The goal was to provide year-round access to living plant material within existing laboratory spaces while remaining feasible for the space, budget, and instructional conditions typical of PUIs (Almerino and Peria, 2024).

### 3.2 Course integration and instructional design

Hydroponic systems were incorporated into upper-level biology courses, including BIO 313–Botany (approximately 15 students per offering) and BIO 342–Mushrooms & Molds (approximately 20 students per offering). With appropriate scaffolding, the framework is also adaptable to introductory biology courses.

The platform was introduced early in the semester and used continuously for approximately 4–8 weeks, allowing students to observe multiple developmental stages. Learning objectives included plant anatomy, physiology, development, responses to environmental stimuli, measurement, data interpretation, and basic engineering concepts related to lighting and water flow (Figure 2).

The instructional framework aligns with CURE design principles, including iterative experimentation, collaboration, data generation, and relevance beyond the classroom (Broussard et al., 2025; Gibbons Johnson 2025). Students modified variables (such as lighting, nutrient composition, and seeding density), analyzed outcomes, and refined approaches over multiple growth cycles (Cummings and Cummings, 2021; Lakomy et al., 2024). Group work and presentations reinforced scientific communication, positioning the hydroponic platform as both a hands-on teaching tool and a structured CURE environment (Bennie et al., 2025; DeChenne-Peters and Scheuermann, 2022).

### 3.3 Physical setup and infrastructure adaptation

Hydroponic systems were installed in an existing teaching laboratory (ARM 212), avoiding new construction or renovation. Commercial vertical farming units (e.g., Babylon V6 farms, Gardyn Towers, Bootstrap Farms systems, and a HarvestWall) were combined with custom Dutch bucket systems with automated water cycling to increase instructional flexibility.

Together, these systems provided approximately 2,700 planting sites within a compact footprint. System selection emphasized modularity, compatibility with available electrical and water access, scalability and cost. Commercial units ranged from several hundred to tens of thousands of dollars, whereas custom (raft- or bucket-based) systems could be built at lower cost. Consumables such as seeds, probes, and nutrient solutions were incorporated into routine course budgets (Guerrero-Sánchez et al., 2025; Prats et al., 2026).

### 3.4 Instructional implementation and student engagement

Students received demonstrations and hands-on training in safe operation, maintenance, and experimental use of the hydroponic systems. They then worked independently or in small groups, typically two to three students, to conduct scaled experiments.

Activities included seed germination and transplanting, nutrient preparation and monitoring, growth observation and phenotyping, and harvest with post-harvest analysis (Figures 1, 2). Students tested variables such as light wavelength and photoperiod, seeding density, substrate type, water cycling, and airflow, while monitoring parameters included pH, electrical conductivity, light intensity, temperature, and growth duration.

Students also helped refill reservoirs and adjust nutrient parameters. These responsibilities fostered ownership and accountability while reinforcing links among experimental design, system management, and biological outcomes (Cummings and Cummings, 2021; Lakomy et al., 2024).

### 3.5 Assessment, outcomes, and pedagogical impact

Student learning was assessed through laboratory reports, inclass or poster presentations, and direct inspection of plant performance. Students generated quantitative datasets, including growth measurements, biomass estimates, seeding density maps, harvest weights, and environmental parameter logs.

Observed outcomes included strong engagement, improved conceptual understanding of plant anatomy and physiology, and increased confidence with controlled-environment cultivation. Rapid plant responses helped students connect experimental decisions and maintenance practices with biological outcomes, reinforcing authentic inquiry.

Living plants in the classroom also strengthened instruction by allowing demonstrations with actively growing specimens rather than only images or preserved materials. Compared with field-based instruction, the controlled environment enabled variable isolation and year-round experimentation, which is especially useful for spring-semester botany courses in Appalachian climates.

Although these outcomes indicate engagement and meaningful learning, formal assessment with validated education research instruments was beyond the scope of this implementation. Reported outcomes are based on course artifacts, instructor observation, and student research outputs. Future studies should use validated assessment surveys, pre-and post-course assessments, reflection analysis, and comparison groups when feasible to evaluate whether hydroponic-based CUREs improve learning, scientific identity, self-efficacy, and STEM persistence compared with traditional laboratories or field-based instruction (Murren et al., 2026; Zhang et al., 2025).

### 3.6 Transferability and sustainability at PUIs

This implementation strategy is transferable to other PUIs with limited infrastructure. The systems described here are modular, space-efficient, and adaptable; essential requirements include reliable electrical and water access, basic environmental monitoring tools, and faculty commitment to setup and training. Maintenance demands are modest and can be incorporated into student responsibilities to support both instruct and sustainability.

Although developed at a rural PUI, the model’s core components-modular systems, inquiry-driven design, and student-managed experimentation - can be adjusted for different enrollments, and disciplinary emphases. Institution can scale the system, plant selection, and assessment approach to local needs while expanding experiential learning and undergraduate researchy without greenhouse infrastructure (Prats et al., 2026; Wu et al., 2025).

## 4 Educational and funding outcomes of hydroponic system integration at a PUI

At UPIKE, hydroponic integration increased engagement in plant- and agriculture-related disciplines, including botany, mushroom cultivation, and biotechnology. Access to year-round controlled-environment systems enabled authentic, student-led research in experimental design, data collection, and scientific communication. During the Fall 2025 semester, undergraduate students presented 17 research posters at college-level and regional conferences, demonstrating scholarly outcomes that are often difficult to achieve at PUIs with limited research infrastructure.

The platform also strengthened institutional competitiveness for external funding by demonstrating research readiness. It has been integrated into CUREs and projects supported by the NSF/KY ERISE II Grant, the USDA Specialty Crop Block Grant, and NIH/KY INBRE. Because hydroponic teaching platforms align with priorities in undergraduate research, workforce development, sustainability, and capacity building, they can support education-focused proposals to NSF, USDA, and NIH, including mechanisms that integrate research into undergraduate curricula.

In our institutional experience, the system also supported competitive submissions to NIH (R15/R16), USDA, and NSF.

## 5 Conclusion

Hydroponic system integration provides a practical and scalable strategy for expanding experiential learning and undergraduate research capacity at PUIs with limited infrastructure. By enabling year-round, controlled-environment cultivation within existing laboratories, hydroponic platforms function as more than demonstrations; they serve as teaching and research infrastructure that supports student engagement, CUREs, and faculty-guided scholarship.

At UPIKE, this approach was associated with increased student engagement and tangible scholarly outputs, including undergraduate conference presentations.

More broadly, hydroponic platforms align with national priorities in inclusive STEM education, workforce preparation, and institutional capacity building by connecting classroom learning to controlled-environment agriculture, plant biotechnology, and sustainable food systems.

This implementation also has limitations. Outcomes were based primarily on course artifacts, instructor observation, student presentations, and institutional research outputs rather than validated education research instruments. Future research should use validated assessment tools and comparative study designs to evaluate long-term effects of hydroponic-based CUREs on student learning, scientific identity, research confidence, and STEM career persistence (Zhang et al., 2025).

Overall, hydroponic teaching platforms occupy a valuable middle ground between outdoor field-based learning and high-cost greenhouse infrastructure. For PUIs seeking to integrate integrate research into undergraduate education, they offer a practical, scalable, and evidence-aligned pathway for inclusive, research-centered STEM instruction (Pennisi et al., 2025).

Annie Wang, Brooke Duncan, Emily Duncan, Eli Johnson, Kevin Wang and Byron Meade

Byron Meade, Annie Wang, Brooke Duncan, Emily Duncan, Eli Johnson, Kevin Wang

## Figures and Tables

**TABLE 1:** Advantages of hydroponic implementation in PUI plant sciences.

Category	Advantages	Impact
Students	Enhanced engagement through cultivation of familiar plant species (Section 2.1); exposure to controlled-environment plant systems (Section 2.2)	Improved conceptual understanding of plant biology; development of experimental, technical, and data-analysis skills; increased interest in plant science–related academic and career pathways
Faculty	Flexible, year-round teaching platform independent of greenhouse access (Section 2.3); reproducible experimental conditions; research funding inquiry	Improved instructional effectiveness; ability to integrate inquiry-based activities into existing courses; alignment of teaching with undergraduate research and scholarly activity
Institution	Scalable use of existing laboratory space; visible commitment to experiential learning (Section 2.4)	Strengthened STEM education capacity; improved student recruitment and retention; enhanced competitiveness for external education and infrastructure funding
